# Established and Emerging Research Trends in Norway Lobster, *Nephrops norvegicus*

**DOI:** 10.3390/biology12020225

**Published:** 2023-01-31

**Authors:** Jacopo Aguzzi, Simona Violino, Corrado Costa, Nixon Bahamon, Joan Navarro, Damianos Chatzievangelou, Nathan J. Robinson, Jennifer Doyle, Michela Martinelli, Colm Lordan, Joan B. Company

**Affiliations:** 1 Instituto de Ciencias del Mar (ICM-CSIC), Consejo Superior de Investigaciones Científicas, 08003 Barcelona, Spain; 2 Stazione Zoologica di Napoli (SZN) Anton Dohrn, 80121 Naples, Italy; 3 Consiglio Per La Ricerca in Agricoltura E L’analisi Dell’economia Agraria (CREA)-Centro Di Ricerca Ingegneria E Trasformazioni Agroalimentari, Via della Pascolare 16, Monterotondo, 00015 Rome, Italy; 4 Marine Institute (MI), Fisheries and Ecosystem Advisory Services, H91 R673 Galway, Ireland; 5 National Research Council−Institute of Marine Biological Resources and Biotechnologies (CNR-IRBIM), Largo Fiera della Pesca 2, 60125 Ancona, Italy

**Keywords:** Norway lobster, burrow emergence, ecological monitoring, stock assessment, ecosystem-based approaches, food, ecotoxicology

## Abstract

**Simple Summary:**

The Norway lobster is a key species for European Atlantic and Mediterranean fisheries. We performed a global bibliographic survey using the VOSviewer software to investigate the status of research on Norway lobsters by extracting data from all relevant scientific literature published in SCOPUS since 1965. The analysis revealed three clusters: (1) fishery performance, stock assessment, and management; (2) biological cycles in growth, reproduction, and behavior; and (3) physiology and ecotoxicology (including food products). Intense research has emerged on ecotoxicology and fishery management (which also includes other commercially targeted and co-existing species). Finally, earlier studies generally focused on the morphological and physiological aspects of this species while more recent studies have focused on fisheries and on Norway lobster as a food resource. In summary, the results indicate that knowledge is scarce on how the burrowing behavior of *N. norvegicus* is influenced by environmental conditions and how this affects stock assessments. We attribute this to limited use of current and advanced monitoring technologies.

**Abstract:**

The burrowing crustacean decapod *Nephrops norvegicus* is a significant species in European Atlantic and Mediterranean fisheries. Research over the decades has mainly focused on behavioral and physiological aspects related to the burrowing lifestyle, since animals can only be captured by trawls when engaged in emergence on the seabed. Here, we performed a global bibliographic survey of all the scientific literature retrieved in SCOPUS since 1965, and terminology maps were produced with the VOSviewer software to reveal established and emerging research areas. We produced three term-map plots: term clustering, term citation, and term year. The term clustering network showed three clusters: fishery performance, assessment, and management; biological cycles in growth, reproduction, and behavior; and finally, physiology and ecotoxicology, including food products. The term citation map showed that intense research is developed on ecotoxicology and fishery management. Finally, the term year map showed that the species was first studied in its morphological and physiological aspects and more recently in relation to fishery and as a food resource. Taken together, the results indicate scarce knowledge on how burrowing behavior and its environmental control can alter stock assessment, because of the poor use of current and advanced monitoring technologies.

## 1. Introduction

The Norway lobster, *Nephrops norvegicus*, is a burrowing decapod that inhabits muddy (i.e., silt and clay) seabeds on the Atlantic and Mediterranean continental shelves and slopes [1,2]. Adult lobsters construct a burrow system of a 20–30 cm depth, with two major crater-like entrances and several lateral ventilator shafts [3,4,5,6,7,8,9,10]. The species is an important pillar of European fisheries, generating an annual value of 107 M€ [11]. In the Atlantic, *N. norvegicus* is the second most valuable landed shellfish species in the North Sea and eastern Arctic regions since 2018 [12]. Its burrowing behavior also increases habitat heterogeneity and provides structures of relevance for co-existing megafauna. Therefore, protecting *N. norvegicus* indirectly protects many other co-occurring fauna, being an umbrella species with additional relevant functions such as ecosystem engineer [13]. At the same time, it is significant prey for diverse demersal predators [14].

Due to its economic and ecological importance, *N. norvegicus* has been the focus of intense scientific research over the past 40 years. Studies have characterized different aspects of its burrowing behavior (activity rhythms affecting trawl availability; see below) and underlying physiological aspects (e.g., its survival tolerance to different environmental factors), along with population dynamics (e.g., density, biomass, distribution, and connectivity of populations) [15,16,17]. Research on the burrowing behavior of *N. norvegicus* is key due to its profound impact on capture rate of trawl fisheries and associated stock assessments (reviewed by Aguzzi et al. [18,19]). In fact, animals are only captured by trawlers when they are outside their burrow systems [20]. Those residing within their burrow or at the entrance (i.e., door-keeping behavior, in which only a portion of the carapace and claws would be visible [21]) avoid haul capture [15,22]. Burrow emergence is strongly influenced by environmental light, as they are crepuscular on the lower shelf (50–200 m) [3,23,24]; and fully diurnal on the slope (200–450) [25]. Seasonal variations in the length of the photoperiod also regulate the reproductive cycle of the species. Egg-bearing females spend less time outside their burrows (to protect their offspring), resulting in overall catch fluctuations over different months in different latitudes [15,22,26].

Some key environmental factors seem to affect individuals’ physiology and epidemiology. The combination of temperature and salinity influences the survival of all marine species [27]. Temperature has an important effect on the developmental rate and physiology of *N. norvegicus* since a notable shift in the larval release can be attributed to warming [28]. Additionally, muscle and cholinolytic necrosis of the cuticle are induced by bacteria, as observable above 13–14 °C [29,30]. Animals can also be exposed to a seasonal pattern of infection by the parasitic dinoflagellate *Hematodinium* [31,32]. This has a significant impact on burrowing behavior since infected animals have reduced motility [33]. Infection also increases metabolic rates and associated energetic demands, meaning that the animal must spend longer outside the burrow in search of food.

Here, we performed a global bibliographic survey of the scientific literature on *N. norvegicus* since 1965 to reveal established and emerging research areas. To address this issue, we used a Term-Map Analysis to synthesize research trends by quantifying the frequency of keywords [34]. Such information is of relevance due to a growing demand for stock management, which requires robust field knowledge of species’ behavior, physiology, and ecological factors affecting the demography of its populations [19,35,36].

## 2. Materials and Methods

All bibliographic records regarding *N. norvegicus* on the Scopus database, starting from 1965 to the 15 August 2022, were identified according to the protocols reported by Costa et al. [37] and Aguzzi et al. [18]. The SCOPUS database was chosen because it contains a wide selection of scientific literature from the natural sciences [38]. Publications were identified using the following keywords and strings: (Norway AND lobster) OR (*Nephrops* AND *norvegicus*) OR (Norwegian AND lobster) in the combined “Title”, “Abstract”, and “KeyWords” fields. To avoid false positives (particularly when different meanings have unique singular and plural forms), the publications obtained from the query were validated by manual screening of the title and, when necessary, of the abstract. Only terms recurring at least ten times were extracted [39].

Bibliometric maps were created using VOSviewer software (version 1.6.16 and available for free at www.vosviewer.com; accessed on 30 January 2023). The software was developed specifically to create, visualize, and explore scientific bibliometric maps [34]; for further explanation of the method see also [39,40].

Firstly, a thesaurus file was created to ensure consistency between different spellings of terms and synonyms (Supplementary Information S1). Terminology maps were created using the method described by Van Eck and Waltman [34]. An EndNote file for this research is included in Supplementary Information S2. The network files needed to navigate the maps with labels are available as Supplementary Information S3 and S4, respectively.

Next, different maps were produced by the VOSviewer software: (1) term clustering, (2) term citation, and (3) term year. The term clustering map depicts the strength of the relationship between two terms, based on how frequently they co-occur within the scientific literature. Terms that co-occur in the same publications are found close to each other in this map, and those that are less strongly related (low co-occurrence) are found farther away from each other, forming conceptual clusters which are visually distinguished by different colors (i.e., red, blue, and green). Each term is identified by a circle, whose diameter and label size indicate the number of publications in which the term appears in the title, abstract, or keywords. The software uses a VOS clustering technique, i.e., a weighted and parameterized variant of modularity-based clustering [41,42]. The term co-occurrence map is associated with a strength that specifies the similarity between the two conceptual ideas and was calculated as follows:

s_ij_ = c_ij_/w_i_w_j_ (1)

where s_ij_ indicates the similarity measure, c_ij_ represents the number of co-occurrences between two conceptual ideas _i_ and _j_, and w_i_w_j_ is the total number of co-occurrences of conceptual ideas _i_ and _j_ [43].

The term citation map analyzes the scientific impact of specific topics, while the term year map performs a temporal analysis of research topics. In detail, in the term citation map, the color of a term is determined by the normalized average citation impact of the publications in which the term appears. The greater the number of publications in which two terms co-occur, the stronger the correlation between the terms. To avoid biases associated with the age of a publication (older publications generally have more citations), the number of citations in each publication is divided by the average number of citations in all publications in the same year. This yields a publication’s normalized citation score, ranging from 0 to 1.5. In the map, colors are assigned according to the score, from blue (average score of 0.8) to green (average score of 1.1) to yellow (average score of 1.4). Thus, a blue (cold) or yellow (hot) term indicates publications in which the term recurs with low and high average citation impact, respectively [44].

In the term year map, the color of a term identifies the average year of publication in all publications in which the term occurs. The colors range from blue (average year of occurrence of the term 2000 or earlier), to green (2005) to yellow (2010 or later). Accordingly, blue terms are those that occur primarily in older publications, while yellow terms occur primarily in more recent publications. To avoid label overlap, only a subset of all labels is displayed in the maps. This map represents results only in the time window ranging from 2000 to 2010. One should consider that the average value is unbalanced, i.e., there is more literature in recent years than in the decade 1960–1970. Therefore, the value is skewed forward, but it represents the average value of when the single term was used.

## 3. Results

**Figure 1:**
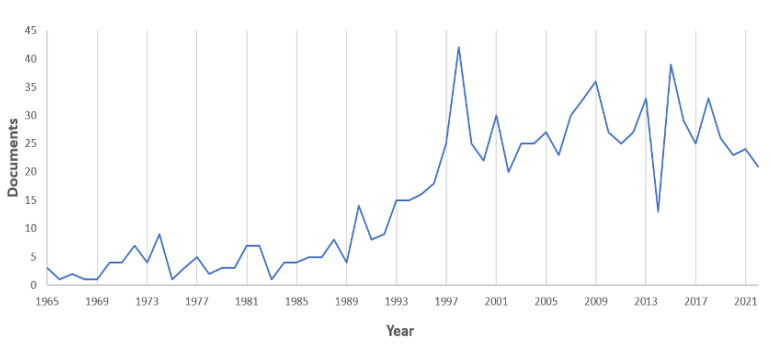
Temporal trend in published papers on *N. norvegicus* showing a sharp increase after 1989 onward. The last year (2022) is not finalized, so the total number of published papers is still incomplete (i.e., runs until the 15 August).

We analyzed 901 scientific publications that provided a combined total of 19,810 terms. An increasing number of publications on *N. norvegicus* were produced from 1965 to 2022 (the last year included in the present study; Figure 1). Interestingly, a sharp rise in the publication rate began in 1989–1990, but after 1999 the number of publications plateaued. This rise can be explained with the establishment of an expert working group on *N. norvegicus* stock assessment within the International Council for Exploration of the Sea (ICES) framework in the late 1980s. This group and its network have driven research projects to address scientific questions through research funding mechanisms since its inception.

803 publications (89.1%) were research papers. The remaining 98 included conference papers (54), reviews (29), and book chapters (15). The top 10 co-author affiliations for all publishing items retrieved, together with the number of documents and the percentage these institutions represent out of the total detected affiliations, are reported in Table 1. Co-authors based in countries within the distribution area of *N. norvegicus* composed 85.9% of papers on this species. European Union co-authors (51.0%) resulted in the top of the list in terms of number of papers, followed by the United Kingdom (27.3%).

### The Areas of Biological and Ecological Research

**Figure 2:**
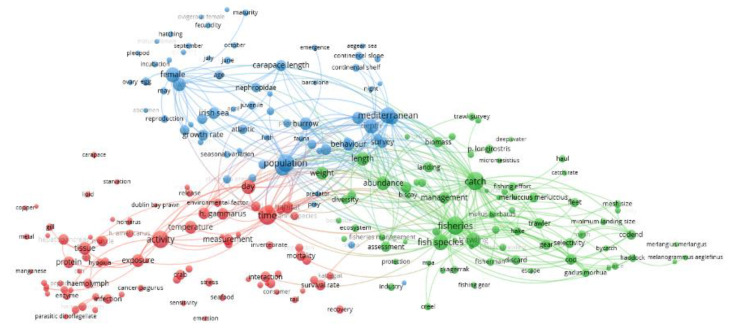
The term clustering map based on the analysis of publications concerned with *N. norvegicus* retrieved from SCOPUS database from the period 1965–2022. Red, blue, and green colors represent the terms belonging to different clusters. The dot size of each term is based on the number of its occurrence. The connecting lines indicate the 300 strongest co-occurrence links between terms.

The term clustering network plot showing closeness among terms was calculated based on term co-occurrence within the same publication. Three clusters emerged from that analysis (Figure 2): fishery performance, stock assessment, and management (green circles—85 terms); biological cycles in growth, reproduction, and behavior (blue circles—69 terms); and finally, physiology and ecotoxicology, including food products (red dots—85 terms).

The green cluster “fishery performance, stock assessment, and management” includes terms related to the commercial harvesting procedure such as “fisheries”, “fish species”, “catch”, “management”, and “trawling”. Those key terms separate the green cluster into two halves: one side with demographic indicators from hauls as “abundance”, “weight”, and “length”, with some relation to “assessment” and “protection”; the other side with boat and net specification-related terms (e.g., “selectivity”, “gear”, “codend”, “escape”, “mesh size”, “discard”, and “bycatch”) and other species as additional targets (e.g., “*Merlangius merlangius*”, “*Melanogrammus aeglefinus*”, “*Gadus morua*”, *“Merluccius merluccius”*, “*P. longirostris*”, “*Mullus barbatus*”, and “haddock”).

The blue cluster “biological cycles in growth, reproduction, and behavior” embraces terms related to the reproductive cycle of “females” (e.g., “hatch”, “fecundity”, “maturity”, “ovary”, “egg”, “incubation”, and “pleopod”) and particular months in the life cycle (“October”, “May”, “July”, and “June”). At the same time, this cluster includes terms related to the growth cycle (“growth rate”, “juvenile”, and “carapace length”), connected to two locations, one general, the “Atlantic”, and the other more specific, the “Irish Sea”. Finally, the portion of this cluster close to the green one (see above) is related to the different aspects likely influencing the stock assessment, such as “burrow”, “behavior”, “survey”, “population”, “fauna”, and “depth”. These latter terms are interestingly connected to “Mediterranean”, “continental shelf”, and “continental slope”. Additionally, the term “video” is the only technological one, appearing close to the bottom part, within the green cluster (see above), disconnected from the rest of the blue cluster elements.

The red cluster “physiology and ecotoxicology, including food products” encompasses a group of characters related to the physiological performance, such as “tissue”, “protein”, “hypoxia”, “haemolymph”, and “enzyme”, with hints of alterations for “infection” by “parasitic dinoflagellate”. Emerging terms are “activity”, “temperature”, and “time” that refer to physiological performances of animals during the fishery process and their response to changing habitat conditions. Sparse terms are also present in relation to food products and their quality (e.g., “seafood”, “consumer”, “survival rate”, “emersion”, and “tail”). Interestingly, the terms “time”, “day”, and “habitat” for this cluster appear well-connected to both “population” (blue cluster) and “fisheries” (green cluster). These are generic terms that seem to be related to the methodologies of research in sampling and data analysis. However, the physiological terms are more disconnected among each other and from those of other clusters, indicating a more punctual and independent research.

When comparing the three clusters together (see Figure 2), one could notice that the green and blue terms have many more linkages among each other and with the terms of other clusters, but less with red ones. This can be considered a proxy of multidisciplinary research activity into green and blue domains. For example, the word "survey" (blue cluster) itself, which is still positioned close to “biomass” and “abundance” (green cluster) but differentiated from "trawl survey" (green cluster), may not seem to be strictly connected to behavioral aspects; however, this probably stands close to the word “behavior”, because in this case it is intended for UnderWater TeleVision (UWTV) surveys. In any case, both types of surveys are associated as witnessed by the central blue terms “behavior” and “populations”, indicating that burrow emergence research is associated with the evaluation of biases in demographic assessment. In contrast, the red cluster has several terms unlinked to other clusters, as an indicator of less multidisciplinary and integrated research. The red terms seem more focused on narrower, more specific research scopes.

**Figure 3:**
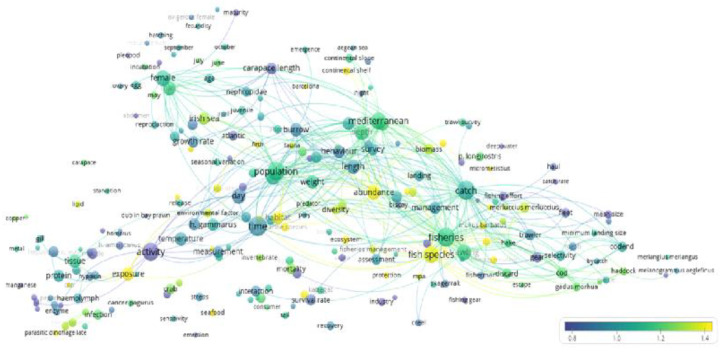
The citation map, indicating the frequency of term citation (i.e., the hotness of topics) within the surveyed bibliography.

The term citation map (Figure 3) indicated the hot research topics by term averaging and normalization (i.e., the most important terms are quantitatively presented per ranking number in Table 2). Intense research is developed on ecotoxicology (i.e., “microplastic”, “ingestion”, and “exposure”, as well as “parasitic dinoflagellate”), with efforts towards creating a context for the community associated with *N. norvegicus* (i.e., an “ecosystem”- and “habitat”-based approach with “management measure” for “protection” in relation to “bottom trawling”), which also includes research on other commercially-targeted “marine species” (i.e., “cephalopods”, “*Mullus barbatus*”, and “*Micromesistius*”). The term "microplastics" is located to the left of the term “Measurements”. These terms are the ones most frequently mentioned. The terms in yellow are those that have the greatest citation impact. Interestingly, the location “Barcelona” appears along with an iconic deep-sea fishery of the “*Aristeus antennatus*” carried out by the same fishing fleets targeting *N. norvegicus*, an indication of common research effort on both items. Interestingly, “seafood” also appears in the ranking of term importance.

Conversely, Figure 3 shows limited research devoted to commercial fish parameters such as “fishing effort” (e.g., “haul”, “catch rate”, trawler”, and “fleet”) and “selectivity” (e.g., “mesh size”, “codend”, and “bycatch”). Similarly, sparse research effort is devoted to behavioral aspects related to burrow emergence (e.g., “day”, “time”, “behavior”, and “burrow”) and the environmental factors (e.g., “hypoxia”) that may alter behavior along with overall catchability. Larger cluster terms (see Figure 2) such as “Mediterranean”, “catch”, “fisheries”, and “population” receive intermediate attention.

**Figure 4:**
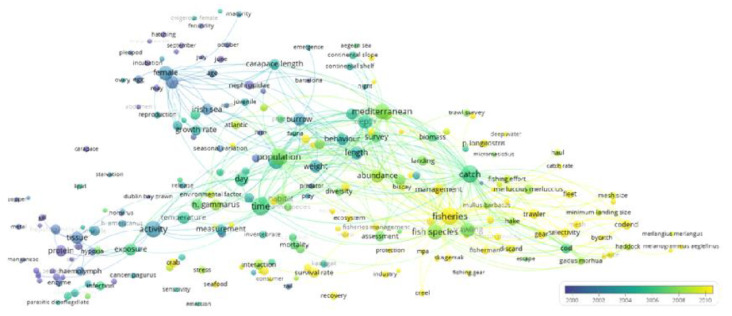
Term year map analysis results, depicting the global temporal trend in the development of research topics.

In the term year map (Figure 4), the color of a term identifies the average year of publication from all publications in which the term occurs. The term-map plot indicates a temporal trend from old to recently used terms, reading from the left (i.e., the blue and red clusters in Figure 2) to the right (i.e., the green cluster in Figure 2), considering that the year values of each term represent the mean. This is as if *N. norvegicus* had been studied first in its morphological and physiological aspects and then, stemming from this research, as an application for fisheries and as a food resource. In particular, more yellow and green (i.e., more recent) terms occur almost entirely in the previously identified “fishery performance, stock assessment, and management” cluster (green in Figure 2). Additionally, this coloring status is also visible in the terms in the inferior part of the previously identified “physiology and ecotoxicology, including food products” cluster (as red in Figure 2). Those terms are very recent (e.g., “seafood”, “industry”, and “consumer”) and, thus, exhibit poor connections with other terms.

## 4. Discussion

The Term-Map Analysis on the *N. norvegicus* literature revealed three major clusters of terms that presently describe the global orientation and status of *N. norvegicus* research: fishery management, biological cycles, and physiology (respectively, green, blue, and red clusters of Figure 2). A comparatively poorer research effort is dedicated to clarifying how burrowing behavior and its environmental control can alter our perception of stock demography (i.e., because of influencing trawl catches or video-based censuses). In fact, the terms related to the burrowing behavior chiefly appear to be sparsely included within the blue cluster. At the same time, the Term-Map Analysis also reveals a scarcity of technological terms. Again, the absence of references to imaging or acoustic tagging is because the species’ burrowing behavior has been poorly monitored in the field. Additionally, no sensors quantifying animals’ responses to a changing seascape have been used in situ, while several key oceanographic factors are considered to be of pivotal importance in modulating populations’ health and trawl haul availability (see next Section 5).

### 4.1. The Research Status

The major lines of research, revealed by the Term-Map Analysis, included fishery management with approaches to trawl net specifications (e.g., mesh size for selectivity) in recent years. Additionally, mentions of growth and reproduction appear along the names of different marine geographic regions. In fact, the terms referring to those aspects fall in the blue cluster (i.e., biological cycles; see Figure 2). Field studies on seasonal patterns of growth and reproduction have been historically driven by certain European institutions, primarily in the Atlantic Ocean (mainly in the Irish Sea; see Table 1). Another group of terms, referring to burrowing behavior and overall catchability, were instead mainly carried out in the northwestern Mediterranean. This indicates the occurrence of two major blocks of action: where mono-specific and industrial fishery occurs (i.e., the Atlantic Ocean and the North Sea), stock assessment practices impose research on fishing practices and technologies; where the fishery is, instead, multi-specific [45], a more diverse character of research occurs, and stock assessment topics are accompanied by studies on behavior.

If *N. norvegicus* research is driven by the traditional lines of research developed at different institutions, it is because there are different established research groups with variable expertise. The local character of studies is also likely a product of variable field support via the cooperation with institutional monitoring programs [46] and local fishery associations, both of pivotal relevance for sampling and data provision. Examples of this are the current management actions based on: UWTV surveys of *Nephrops* Functional Units (FUs; [47]); the Mediterranean International Trawl Survey (MEDITS) program [48]; the recent creation of a network of deep-sea fishing no-take areas (Spanish State Official Bulletin, BOE-A-2022-13834), co-managed with fishers for the restoration of *N. norvegicus* stocks in the northwestern Mediterranean [35]; and, finally, the monitoring program of the Pomo Pits permanent Fishery Restricted Area (FRA) in the Adriatic Sea, established for the protection of essential fish habitats of demersal stocks [49,50]. The latter two strategies fall within the current policy of limiting trawling impacts, recovering the species through the establishment of no-take areas [50,51,52].

### 4.2. A Conservation-Oriented Fishery Research

The analysis of temporal trends of research topics (see Figure 4) indicates a progressive shift in focus over the years. At the beginning, the research on *N. norvegicus* was focused on physiology, biology, and behavior, whereas, in the last years, the research progressively shifted toward fishery management themes. This observation is also supported by the higher normalized citation rate (see Figure 3) for terms within the clusters for fishery management and biological cycles (respectively, green and blue clusters of Figure 2). *N. norvegicus* stocks are showing signs of decline in the Atlantic [53,54] and Mediterranean [55].

An indicator of fishery overexploitation is represented by the reduction in harvested biomass, because of fewer individuals and/or smaller sizes [56]. For example, in the NW Mediterranean, *N. norvegicus* stock biomass is decreasing at a slower pace compared to other co-inhabiting demersal resources (e.g., the European hake, *Merluccius merluccius*) [57]. Some sort of burrow emergence-related dynamics may have helped slow down fishery overexploitation effects, *N. norvegicus* being differently exposed to the threat of trawling (i.e., catchability) in comparison to other species [58]. A proportion of the population may not emerge on a diel basis, its emergence being modulated upon contingent and poorly known ecological factors (also see next Section 5).

### 4.3. A Traditional Stock Assessment with No New Technological Components

A lack of use of in situ stock monitoring technologies is shown by our Term-Map Analysis (see Figure 3 and Supplementary Material S1), despite the growing of those technologies in marine ecology [59,60]. Individual and population tracking technologies could be used to clarify how intra- and interspecific competition (e.g., territorial interactions, predators, and prey presence, as well as the hunger state) modulate the propensity of animals to emerge from their burrows over the 24 h, producing day–night and seasonal fluctuations in captures (e.g., reported drops in catches in spring-summer are due to females sheltering for an egg-bearing condition [26]).

A key aspect of a new technologically sustained approach to the monitoring of burrowing behavior would be the implementation of 4D data collection strategies, i.e., a benthopelagic 3D coverage of the water column plus the seabed, in a temporally intensive fashion (at a frequency of minutes over consecutive days, for several months up to the year) by networks of spatially arranged stationary (e.g., landers) and mobile robotic platforms (lander-docked crawlers and AUVs) [61,62,63].

The need to incorporate the above-mentioned technologies is documented by the ICES expert Working Group on *Nephrops* Surveys [47]. Imaging equipment could be deployed along with other behavioral tracking technologies such as acoustic telemetry [64,65], whose reference is missing from the Term-Map Analysis, despite their increasing relevance in behavioral ecology [66,67]. The active acoustic tracking of emitting individuals could be used to strategically quantify the repetition and duration of door-keeping and emergence phases in individuals at certain burrows. By replicating this monitoring approach for several individuals (enough to be considered representative of a population) and burrow systems, the fishery-independent stock assessment could be enhanced [19]; field behavioral data on the occupation and use of burrows could be compared with UWTV surveys detailing stock sizes by burrow counting alone, based on the stock equation “1 burrow—1 animal”.

### 4.4. The Status of Physiological Research

The Term-Map Analysis indicated a separation of the physiological research from all other branches (low connections of the red cluster extreme terms with the blue and green clusters in Figure 2). Physiological research is detached from the other clusters because it has been performed mainly in a lab, after all. *N. norvegicus* physiological research has been traditionally carried out in relation to the themes of hormonal regulation of growth and reproduction [68,69,70]. Moreover, research has also been extensively carried out in relation to the eye structure and damage to light exposure [71,72,73,74,75].

Presently, physiological research in the field with in situ monitoring technologies (see previous Section 4) would disclose relevant information on the species’ tolerance to the ranges of variation in key oceanographic variables such as temperature and salinity. Water warming and salinity changes produce shafts in species distributions because of their direct effects on species metabolism [76,77,78]. For *N. norvegicus*, established research exists on those effects at the level of growth, reproduction, immunological responses, and overall survival [79,80,81]. Slight variations in salinity ranges also showed potential impacts on the species abundance and local distribution [82].

Conversely, little research exists in relation to dissolved oxygen concentration [83,84] as a mechanical explanation for variations in haul catches, i.e., animals in anoxic eutrophic grounds spend more time out of the burrows to ventilate and are more prone to net capture with consequent landing peaks. Low levels of oxygen saturation may actually also have negative effects on recruitment [82] as well as salinity stress conditions [81]. Since anoxic conditions and dense waters might be modulated by water mass renewals and current speed, in situ current meters could disclose relevant information on burrow emergence modulation. Those sensors would also be useful for picturing flow variations as light-independent cues tuning the timing of burrow emergence (in turn, impacting catchability) [15].

Other factors that would deserve an in situ tracking of burrow emergence modulation (and overall catchability) would be dissolved carbon anhydride and resulting pH. Ocean acidification [85] alters the physiology, growth, reproduction, sensory perception, behavior, and, in severe instances, the survival of marine species [85,86]. Species may react with geographic distribution shifts, which may produce consequent shifts in the functioning of marine ecosystems [87]. Although crustaceans have higher tolerance to acidification [88], recent studies suggest that juveniles could be particularly sensitive to acidification, especially in combination with other global change drivers [89]. Laboratory experiments have been carried out to assess the sensitivity of *N. norvegicus* to acidification [81,82,83,84,85,86,87,88,89,90]; however, broader field observations are still needed for a more complete understanding of hypercapnic stress effects on this species and general consequences on its populations. Recently developed pH sensors for the high temporal resolution of ocean acidification [91] could be used on permanent platforms such as cabled observatories [61,63] or be deployed along with traditional CTD (conductivity, temperature, and depth) and/or DO (dissolved oxygen) oceanographic probes while carrying out monitoring activities, as it currently happens in various *N. norvegicus* grounds [47,82].

Other important aspects of ecosystem functioning, such as phytoplankton biomass production, may be of concern for *N. norvegicus* physiological research, but it is presently not fully considered, as shown by the research panorama. Santana et al. [92] highlighted the importance of suspended organic particulate matter in the diet of this species, with more suspension feeding in small or medium-sized individuals than larger individuals. Chlorophyll fluorescence sensors may help add this indicator of ecosystem functioning to fishery management, adding an energy dimension to stock productivity. Moreover, river runoff and trawling activity resuspending sediment [93] may have relevant effects on the remineralization processes within the first seabed layers, where *N. norvegicus* dwells and digs its tunnels. Historical trawling activity acts as a geological-like shaping force, flattening continental shelves and slopes, based on sediment removal [94].

The effect of water turbidity (as proxy for dissolved energy/matter) could be measured in situ by other fluorescence sensors, to determine seabed conditions in relation to the health status of the stocks. However, the broad use of oceanographic sensors may not only help understand the species abundance and distribution constraints, but it would also provide innovative multiparametric data for modeling (i.e., covariates help understand and describe the distribution) [82,95].

Accounting for physiological reactions with conventional in situ methodologies has been very challenging, thus limiting physiological research to lab-based experiments. However, newly introduced lab-on-a-chip approaches may assist with the microfluidic detection of hormones, elucidating physiological responses to the changing environment in the near future [96].

### 4.5. The Food Industry as Emerging Research Involving Ecotoxicology

Research on food technology with aspects on food conservation is emerging. The group of terms related to lobster, such as food product, namely “food product”, “quality”, and “consumer”, are closely related to “quality” aspects, including concerns about “microplastic”, but are overall still poorly connected within the red cluster (see Figure 2). As reported by Katoh et al. [17], *N. norvegicus* represents one of the most commercially important food fish products in Europe. To preserve and monitor its quality, it would be necessary to verify the supply chain for *N. norvegicus*, starting with the fresh product, to ensure a quality product for the consumer. In this sense, traceability is essential [97]. The traceability of seafood products is a critical aspect that is supportive of fishery management and consumer protection [98].

Although *N. norvegicus* is a significant economic resource in fisheries, there may be some pollutants that can spoil this product, making it inedible and altering its preservation [99]. In fact, in our work, the most frequently mentioned term is “microplastic”. To date, microplastic is increasingly present in marine litter. In the case of *N. norvegicus* from the North Atlantic and the western Mediterranean, it has been shown that it is able to ingest large amounts of microplastic over a prolonged period of time, which adversely affects the crustacean’s life span and, consequently, its quality [100,101] and that of the whole marine food web. Cau et al. [102] even demonstrated that the characteristic structure of the *N. norvegicus* stomach may have a role in the breakdown of ingested plastic particles into smaller microplastics, which are then released in the intestine and, subsequently, in the environment. Little work has been done thus far on the transfer of microplastics from the digestive tract to other organs, accumulation in tissues, and transfer to the food web [103]. Furthermore, *N. norvegicus* has been recently proposed as a potential monitoring tool for microplastics within the seafloor sediment [104].

## 5. Conclusions

We detected three major research clusters for *N. norvegicus*: “fishery performance, stock assessment, and management”, “biological cycles of growth, reproduction, and behavior “, and “physiology, ecotoxicology, and use as a food product”. Of these, the first cluster on fishery management includes more recent publications. This is likely because increasing stock demand alongside population declines are stimulating research on commercial harvesting procedures (i.e., boat and net specifications). Furthermore, research efforts appear geographically distinct, with Atlantic and North Sea institutions focusing research efforts on demography, growth, and reproduction cycles because of their industrial and mono-specific fishery-oriented approach. In contrast, research in the Mediterranean is more focused on behavior, due to the multispecies approach of the fisheries. Nevertheless, in both cases, more research work is still required to a build better picture of the ecological niche of the species. Specifically, there is a lack of detailed information on the relationship between stock status and environmental conditions. This could be addressed by using innovative sensor packages for ecological monitoring that couple HD and optoacoustic imaging (to record animal behavior and distribution) with oceanographic and geochemical sensors (to characterize environmental conditions affecting burrow emergence and, hence, stock assessment). If implemented, this technology would vastly increase the scope of data available for management and conservation.

## Figures and Tables

**Table 1:** Top 10 institutional affiliations of the papers’ co-authors and pertaining countries, plus the relative percentages out of the total (of all institutional affiliations for all retrieved papers).

Affiliation	Country	Documents	%
Instituto de Ciencias del Mar (ICM-CSIC)	Spain	80	5.3
University of Glasgow	UK	74	4.9
Havforskningsinstituttet	Norway	62	4.1
Marine Scotland Information (MSI; Marine and Fisheries of the Scottish Government)	UK	45	3.0
Göteborgs Universitet	Sweden	43	2.8
Technical University of Denmark	Denmark	42	2.8
University Marine Biological Station Milliport	UK	40	2.6
Centre for the Environment Fisheries and Aquaculture Science	UK	37	2.4
Instituto Español de Oceanografía (IEO)	Spain	34	2.2
Consiglio Nazionale delle Ricerche (CNR)	Italy	34	2.2

**Table 2:** The first 20 hot topic terms (i.e., with the highest citation score), sparse within the term score (average normalized citation) map of Figure 3. Some of those terms are not visible in the map due to labels overlapping, and therefore their positioning has been identified close to other visible terms of reference, with an indication of the belonging cluster (as per Figure 2).

Term	Score	Close to (or Visible)	Into the Cluster
microplastic	39.842	to "measurement"	RED
ingestion	24.547	to "release"	RED
ecosystem	19.063	VISIBLE	GREEN
stomach	18.964	to "growth rate"	BLUE
marine species	18.117	VISIBLE	RED
cephalopod	16.703	among "fisheries", "fish species" and "trawling"	GREEN
*Mullus barbatus*	16.214	VISIBLE	GREEN
*Micromesistius*	15.859	VISIBLE	GREEN
commercial species	15.027	right side of "fisheries" and "trawling"	GREEN
habitat	14.838	VISIBLE	RED
exposure	14.799	VISIBLE	RED
bottom trawling	14.685	VISIBLE	GREEN
management measure	14.408	right side of "catch" and "management"	GREEN
Barcelona	14.152	VISIBLE	BLUE
*Aristeus antennatus*	14.139	right side of "biomass"	GREEN
continental shelf	14.088	VISIBLE	BLUE
seafood	13.925	VISIBLE	RED
protection	13.852	VISIBLE	GREEN
parasitic dinoflagellate	13.807	VISIBLE	RED
abundance	13.801	VISIBLE	GREEN

## Data Availability

Not applicable.

## Supplementary Materials

The following supporting information can be downloaded at: https://www.mdpi.com/article/10.3390/biology12020225/s1, Supplementary Information S1: Thesaurus of terms; Supplementary Information S2: List of extracted references; Supplementary Information S3: Term-Map results; Supplementary Information S4: Term-Map network.
